# Sex Detection of Chicks Based on Audio Technology and Deep Learning Methods

**DOI:** 10.3390/ani12223106

**Published:** 2022-11-10

**Authors:** Zeying Li, Tiemin Zhang, Kaixuan Cuan, Cheng Fang, Hongzhi Zhao, Chenxi Guan, Qilian Yang, Hao Qu

**Affiliations:** 1 College of Engineering, South China Agricultural University, Guangzhou 510642, China; 2 National Engineering Research Center for Breeding Swine Industry, Guangzhou 510642, China; 3 Guangdong Laboratory for Lingnan Modern Agriculture, Guangzhou 510642, China; 4 Institute of Animal Science, Guangdong Academy of Agricultural Sciences, Guangzhou 510642, China

**Keywords:** poultry, chick sexing, audio technology, deep learning method

## Abstract

**Simple Summary:**

Poultry farming is an important part of agriculture production. The automatic sex detection of chicks can help to improve production efficiency and commercial benefits as well as protecting animal welfare. In this study, a chick sexing method is designed, which achieves the automatic sexing of chicks by audio technology and deep learning methods. The experimental results show that this method can detect the sex of chicks by their calls. The chick sexing method designed in this study provides a new means for smart poultry production and can help poultry researchers in the future.

**Abstract:**

The sex detection of chicks is an important work in poultry breeding. Separating chicks of different sexes early can effectively improve production efficiency and commercial benefits. In this paper, based on the difference in calls among one-day-old chicks of different sexes, a sex detection method based on chick calls is designed. Deep learning methods were used to classify the calls of chicks and detect their sex. This experiment studies three different varieties of chicks. The short-time zero-crossing rate was used to automatically detect the endpoints of chick calls in audio. Three kinds of audio features were compared: Spectrogram, Cepstrogram and MFCC+Logfbank. The features were used as the input in neural networks, and there were five kinds of neural networks: CNN, GRU, CRNN, TwoStream and ResNet-50. After the cross-comparison experiment of different varieties of chicks, audio features and neural networks, the ResNet-50 neural network trained with the MFCC+Logfbank audio features of three yellow chick calls had the highest test accuracy of 83% when testing Three-yellow chicks’ calls. The GRU neural network trained with the Spectrogram audio features of native chick calls had the highest test accuracy of 76.8% when testing Native chicks’ calls. The ResNet-50 neural network trained with Spectrogram audio features of flaxen-yellow chick calls had the highest test accuracy of 66.56%when testing flaxen-yellow chick calls. Multiple calls of each chick were detected, and the majority voting method was used to detect the sex of the chicks. The ResNet-50 neural network trained with the Spectrogram of three yellow chick calls had the highest sex detection accuracy of 95% when detecting the three yellow chicks’ sex. The GRU neural network trained with the Spectrogram and cepstrogram of native chick calls and the CRNN network trained with the Spectrogram of native chick calls had the highest sex detection accuracy of 90% when detecting the native chicks’ sex. The Twostream neural network trained with MFCC+Logfbank of flaxen-yellow chick calls and the ResNet-50 network trained with the Spectrogram of flaxen-yellow chick calls had the highest sex detection accuracy of 80% when detecting the flaxen-yellow chicks’ sex. The results of the cross-comparison experiment show that there is a large diversity between the sex differences in chick calls of different breeds. The method is more applicable to chick sex detection in three yellow chicks and less so in native chicks and flaxen-yellow chicks. Additionally, when detecting the sex of chicks of a similar breed to the training chicks, the method obtained better results, while detecting the sex of chicks of other breeds, the detection accuracy was significantly reduced. This paper provides further perspectives on the sex detection method of chicks based on their calls and help and guidance for future research.

## 1. Introduction

In the modern chicken breeding industry, the sex detection of chicks is a very important task. Whether it is breeder, broiler or layer, the feeding and marketing strategies of chickens of different sexes are different. Separating chicks of different sexes early can effectively improve production efficiency and commercial benefits [1,2,3,4]. At present, the widely used methods for sex detection of chicks in the breeding industry include cloaca detection (also known as “vent sexing”), sex-linked trait detection and DNA detection. However, the above methods have their shortcomings in the actual production. The cloaca detection method is highly dependent on the detection skills and experience of detection personnel, and it is an invasive contact operation that may also lead to the disability or death of chicks, damaging animal welfare [5]. The sex-linked trait detection method can only be used for specific chick breeds, which has high requirements for breeding technology and is difficult to be applied quickly [6]. The DNA detection method has very high equipment requirements and costs, which are not suitable for mass production [7].

As one of the important ways for chickens to transmit information, calls contain a lot of information related to chickens. The body and health status of a chicken can be inferred from its calls. Alex and Joseph extracted a variety of audio features, such as Mel frequency cepstral coefficients (MFCC), from chicken’s calls, used a variety of machine learning algorithms, such as k-nearest neighbors, as classifiers to classify chickens’ calls, and judged whether the chicken was sick, with the highest accuracy of 95.1% [8]. Huang et al. extracted the MFCC features of calls made by five-week-old specific pathogen-free (SPF) white leghorn chicken flock and used support vector machine (SVM) as classifier to judge whether chickens were infected with avian influenza, with an accuracy of 84~90% [9]. Cuan et al. calculated the spectrogram and MFCC of calls made by five-week-old specific pathogen-free (SPF) white leghorn chicken flock, used CNN to classify the chickens’ calls, and judged whether the chicken was infected with avian influenza, with the highest accuracy of 97.43% [10]. Du et al. extracted nine audio features from calls made by a 18- to 20-week-old Jingfen hen flock reared in a perch husbandry system based on sound source-filter theory, used support vector machine as classifier to classify calls’ type (such as gakel, alarm and squawk), and judged the thermal comfort of the flock by the number of alarm and squawk sounds, obtaining a sensitivity of 95.1 ± 4.3% and an accuracy of 97.6 ± 1.9% [11]. Mahdavian et al. calculated the peak prominence, short-term energy (STE) and Wiener entropy (WE) of calls made by 120 chicks of two genotypes of Ross and Cobb broilers reared in a standard (commercial poultry farming protocol) environment as audio features and studied the accuracy of chickens’ call detection of different ages and diseases [12]. Herborn et al. extracted 13 audio features, including power spectrum and spectral entropy, from chickens’ calls and calculated the call rate of chickens through Spearman correlation, random forest and linear hybrid models; the calls were collected from 12 commercial Ross 308 mixed-sex flocks (25,090–26,510 chicks placed per flock) [13]. Huang et al. proposed three PV-net neural networks for chicken sound classification, and the highest recognition rate of pecking sound made by 25-day-old white leghorn chicken flock reached 96% [14]. Cuan et al. extracted four audio features, including Logfbank, MFCC, MFCC Delta and MFCC delta delta, from calls made by a five-week-old specific-pathogen-free (SPF) white leghorn chicken flock. The five neural networks CRNN, CNN, LSTM, Bi-LSTM and Transformer were used as classifiers to classify the chickens’ calls. The highest accuracy in the judgement of whether chickens were infected with Newcastle disease was 98.50% [15]. 

Due to the differences in vocal organs in chickens of different sexes, their calls also have different characteristics. Therefore, the analysis of calls can be used to detect the sex of chickens. Pereira et al. recorded one-day-old chick calls in a semi-anechoic chamber with the dimensions of 90 cm long and 50 cm wide and 40 cm high. The temperature inside the chamber was withing the threshold of 17–18 °C in order to expose the birds to cold stress and induce them to vocalize. Then, the pitch frequency, call intensity, first formant frequency and second formant frequency of the calls were calculated. Through a Student’s t-test and Fisher’s test, it was found that the second formant frequency of the chicks’ calls was highly correlated with the sex of chicks [16]. Chen et al. used the auto-correlation method to extract the pitch frequency of chickens’ calls, used the hidden Markov model as a classifier to classify chickens’ calls, and used the majority voting strategy to determine the sex of the chickens, with an average accuracy of 85.41% [17]. Sadeghi and Banakar placed all one-day-old Ross 380 chicks in separate boxes alone for 5 min to minimize stress, and then recorded their vocalizations [18]. A total of 25 audio features were extracted from chicks’ calls. The improved distance evaluation method was used to select features. The support vector machine with Gaussian radial basis function was used as a classifier to classify chickens’ calls. The sex of chicks was judged by chickens’ calls, and the highest accuracy reached 90.74% [18]. Cuan et al. recorded 300 one-day-old yellow-feathered chick calls one by one in a soundproof box for two minutes. Then, they used the MFCC of the chicks’ vocalizations, deep learning methods and the majority voting method to detect chick sex, and the highest accuracy reached 91.25% when using CNN [19].

The paper introduced above studied the calls of chicks of different sexes and detected the sex of the calls. However, the above research has some defects, for example, the number of chickens’ calls is insufficient, and the data lack universality. Only the sex detection of calls was carried out, and the sex detection of chicks was not carried out. Some of the calls in the training set and testing set originated from same chicks, causing false highs in the final results. Only one breed of chicks was used in the experiment, and the persuasion on the applicability of the method is therefore insufficient. To achieve the automatic sex detection of chicks based on calls, aiming to solve the above problems, this paper conducts a more systematic experiment, comparing the calls of different varieties of chicks and detecting the sex of chicks’ calls through different audio features and neural networks. Finally, the sex of each chick is judged by the majority voting method for the call detection results of each chick. This paper introduces a sex detection method of chick based on audio technology and deep learning methods, which can automatically detect chick calls and extract their audio features, and then uses deep learning methods to classify chick calls and detect chick sex. By comparing a variety of audio features and neural networks, and selecting the optimal combination, the detection method with best performance is selected.

## 2. Materials and Methods

### 2.1. Experimental Setup

**Figure 1:**
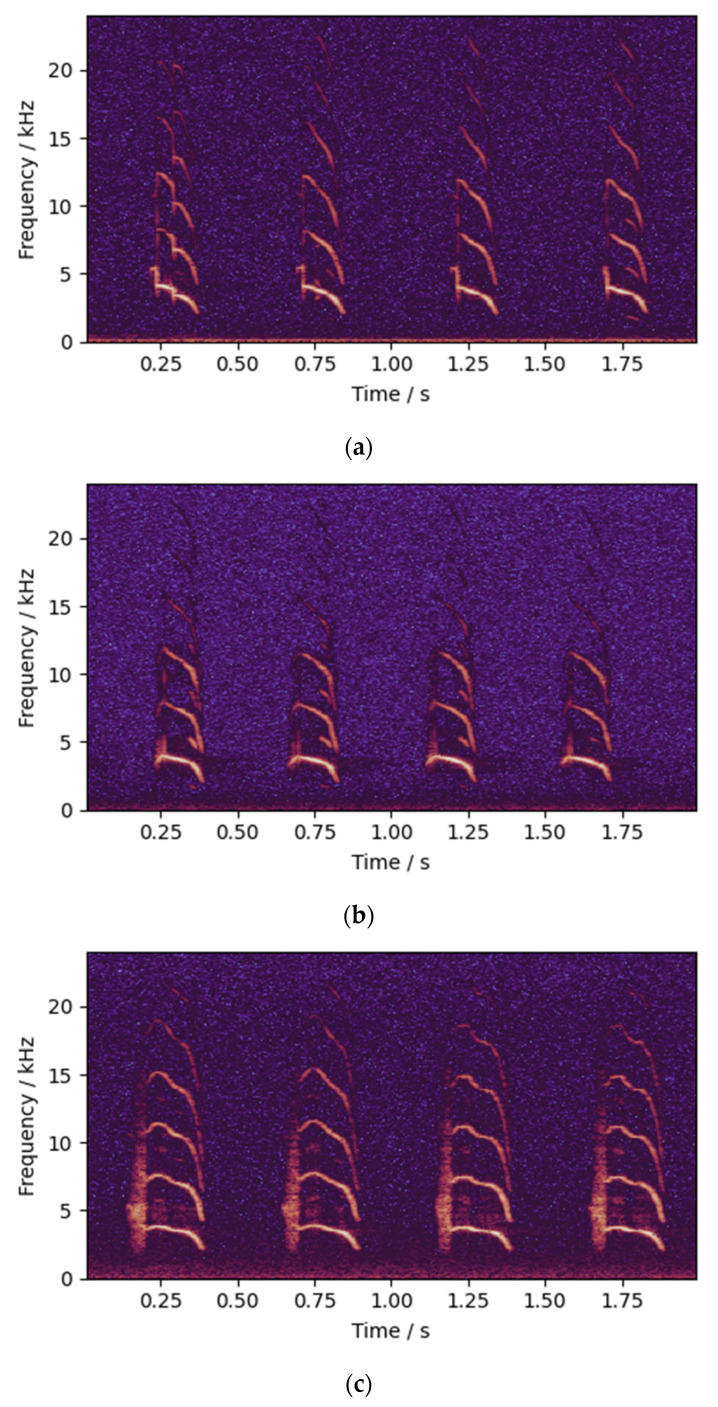
Spectrogram of chicks’ calls: (**a**) three yellow chicken; (**b**) flaxen-yellow chicken; (**c**) and native chicken.

The data used in this experiment were collected from a local hatchery (Guangdong Wiz Agricultural Science & Technology Co., Ltd., Guangzhou, China). A total of 560 chicks were used in the experiment, including 150 cocks and hens of three yellow chickens (TCs), 100 cocks and hens of flaxen-yellow chickens (FCs) and 30 cocks and hens of native chickens (NCs). The three breeds of chicks are slightly different in appearance and physiological characteristics, while their habits are basically the same. All chicks were zero-day-old in age and audio was acquired within hours after their incubation. When chicks are in flocks, the calls are weak and irregular, and the types of calls are diverse. When chicks are isolated from the flock and placed into the data collection system alone, chicks become nervous and stressed and will call for rescue. These calls have high energy, high quantity, and are relatively regular and easy to analyze, and it has been proven that they can be used for sex detection [19], so this kind of call was used as the research object. The spectrograms of the calls of the chicks are shown in Figure 1. The sex of the chicks was determined by professional sorting personnel according to cloaca examination results.

**Figure 2:**
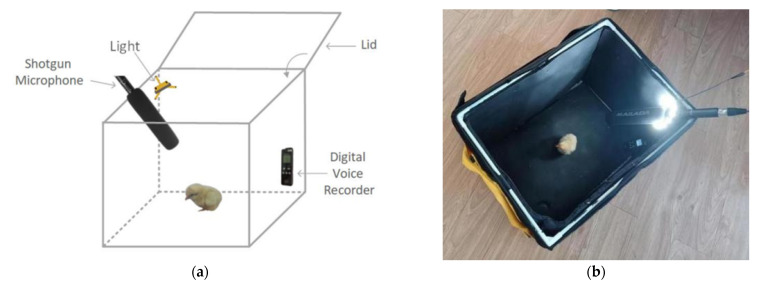
Data collection system: (**a**) diagram; and (**b**) entity.

The main body of the experimental data collection system is a sound-isolating box with a length of 50 cm, a width of 38 cm and a height of 38 cm. The sound-isolating box uses glass fiber rods as support, and the box materials from the outer layer to the inner layer are Oxford cloth, pearl cotton, aluminum foil and sound-absorbing cotton. Pearl cotton and aluminum foil can maintain the internal temperature and prevent chicks from becoming cold. The sound-absorbing cotton can isolate external noise and absorb internal echo. The warm white light LED array was used as the light source in the sound-insulation box to place the chicks in a bright environment similar to the outside world. The recording device was a Lenovo T505 digital voice recorder, and the backup recording device was a MAILADA VG3 shotgun microphone. The recording has a sampling rate of 48,000 Hz and a bit depth of 16-bit. In the experiment, 560 chicks were divided into six groups according to different varieties and sexes. The chicks in each group were separately placed in the sound-insulation box one by one to collect the sound data for 2 min. When waiting to enter the sound-insulation box, the chicks have the supply of special feed and warm water, and the ambient temperature maintained by a heating lamp. They stay in the same wide and comfortable environment with other waiting chicks. The only problem is that the chicks will be isolated from the flock during the experiment, which will lead to mental tension and stress. However, the experiment lasts only 2 min for each chicken, and they are then returned to the flock. During the collection process, the sound-insulation box and the other chicks were placed in different rooms to reduce the impact of other sounds on the chicks. The data collection system is shown in Figure 2.

**Figure 3:**
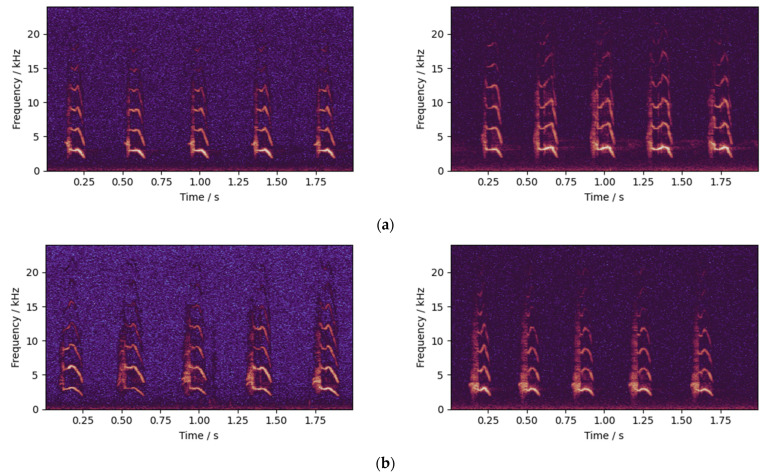
Normalized spectrogram of calls of 2 cocks and 2 hens: (**a**) cocks; and (**b**) hens.

There is no obvious difference in the calls of chicks of different sexes, making it difficult to distinguish different sexes manually. Figure 3 is the normalized spectrogram of the calls of two cocks and two hens. It can be concluded that the cocks and hens’ calls cannot be directly distinguished by human hearing and the observation of spectrogram. Therefore, this paper used audio feature extraction and deep learning methods with their powerful data mining abilities to classify chicken calls.

### 2.2. Pre-Processing

#### 2.2.1. Noise Reduction

In the process of audio acquisition, the recording equipment will collect some additional noise, such as the sound generated by chicks’ walking and people operation. These noises are usually low-frequency sound below 1000 Hz, while the chick’s call frequency is higher, above 1500 Hz. In order to reduce the impact of these noises, high pass filter can be used to denoise the original audio. Infinite impulse response (IIR) filter requires less storage space, less computation, has high efficiency and is easier to design in computer [20], so it was adopted in this paper. The difference equation of IIR filter is shown in Equation (1), where *y*(*n*) is the output value of the filter, *x*(*n*) is the input value of the filter, *n* is the order of the filter, and *a_i_* and *b_j_* are the coefficients of the filter’s system transfer function.

(1) $$y\left(n\right)=\overset{N}{\underset{i\;=1}{\sum }}a_{i}\times y\left(n-1\right)+\overset{N}{\underset{j=0}{\sum }}b_{j}\times x\left(n-j\right)$$

**Figure 4:**
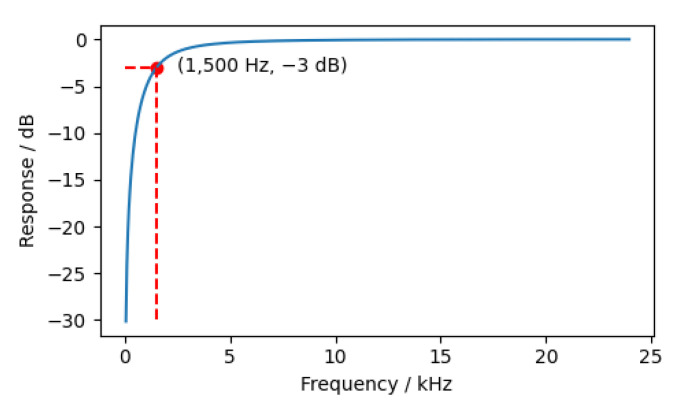
The frequency response of the filter.

In this experiment, a Butterworth high pass filter was constructed using the Python-based SciPy tool. Its −3db frequency was 1500 Hz. The frequency response of the filter is shown in Figure 4.

**Figure 5:**
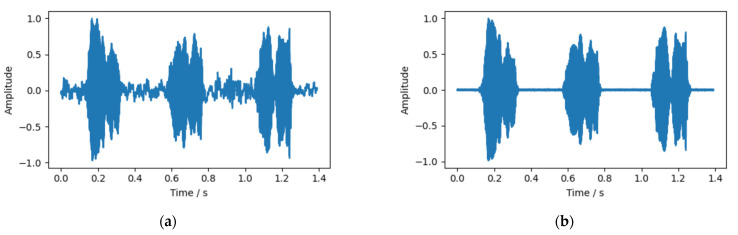
Audio waveform: (**a**) before filtering; and (**b**) after filtering.

The audio after noise reduction by high pass filter and the audio before noise reduction are shown in Figure 5. It can be seen that the chick’s call with higher frequency is not affected too much after filtering, while the noise and clutter with lower frequency are filtered out during filtering.

#### 2.2.2. Framing and Windowing

Chicks’ calls are time-varying. The direct analysis of the origin audio waveform reflects the state of the audio at each time point with difficulty. Therefore, it is necessary to break down the origin audio into short segments by framing, and it is generally considered that sound signals with a duration of 10–30 ms is relatively stable [21]. When the audio is divided into frames, the sudden change in amplitude at the truncated part will produce spectrum leakage. In order to reduce the negative influence of spectrum leakage, the window function can be used to divide the frames. We compared the Hamming window and Hanning window commonly used in audio processing [22]. The attenuation of the Hamming window and Hanning window is shown in Equations (2) and (3), where *M* is the window length.

(2) $$w\left(n\right)=0.54-0.46\times \cos\left(\frac{2\pi n}{M}\right)$$

(3) $$w\left(n\right)=0.5-0.5\times \cos\left(\frac{2\pi n}{M}\right)$$

One of the major differences between Hamming window and Hanning window is the decay at the edge: the Hamming window to 0.08 and the Hanning window to 0. Thus, the Hanning windows performs better in suppressing spectrum leakage.

**Figure 6:**
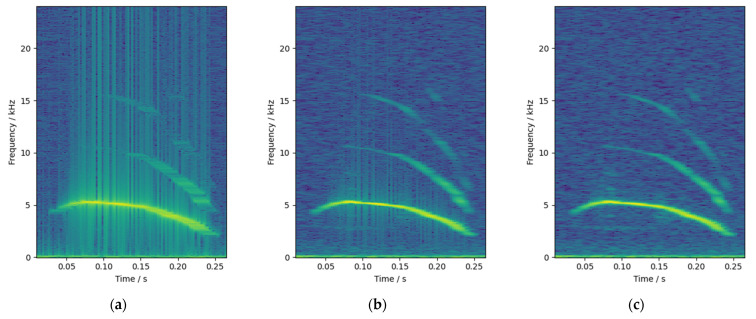
Spectrogram comparison: (**a**) without window; (**b**) Hamming window; and (**c**) Hanning window.

The window function used in this study had a window length of 21.3 ms, including 1024 sampling points, and the step length was 1/8 of the window length, that is, 128 sampling points. As shown in Figure 6, comparing the spectrogram of chicks’ calls without windowing, with Hamming window and with Hanning window, it can be seen that there are considerable burrs in the spectrogram without windowing in Figure 6a, and the situation with Hamming windowing in Figure 6b is significantly improved. In Figure 6c, the burrs are completely removed, and the spectrogram of the audio is clearer and more accurate. Therefore, the Hanning window was selected for windowing and framing in this paper.

### 2.3. Vocalization Endpoint Detection

The audio collected by the recording equipment contains not only the chicken’s calls, but also other sounds and a large number of silent segments. In order to reduce the size of the data, improve the signal-to-noise ratio and improve the automation of the system, it was necessary to use the endpoint detection method to extract the chicks’ calls from the audio. There are various methods of sound endpoint detection. Because the sound environment of chicks’ call collection was clean, the noise impact was small and most of them were low-frequency noises, this paper used an endpoint detection method based on short-time zero crossing rate to extract chicks’ calls from audio. 

Short-time zero-crossing rate refers to the frequency of sound signal changing between positive and negative values in a short time, that is, the number of times the sound waveform passes through the zero value per unit time. It can represent the frequency information of a sound to a certain extent. The higher the main frequency of the sound, the higher the zero-crossing rate. Because the signal usually carries current noise with very high frequency and small amplitude in the process of sound acquisition, it is necessary to set the offset threshold when calculating the true zero-crossing rate. 

Call duration is an important time-domain feature of sound. The duration of different sound events is different. According to the duration of sound, different sound events can be distinguished. The duration of chicks’ calls is short, usually between 0.1–0.35 s, that is, 30 to 110 frames. 

**Figure 7:**
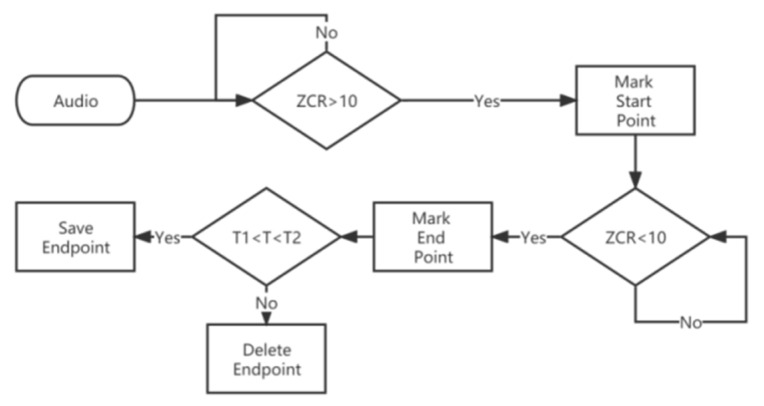
Short-time zero crossing rate endpoint detection method.

**Figure 8:**
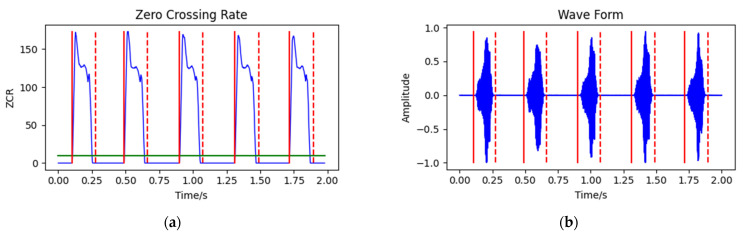
Endpoint detection results of short-time zero-crossing rate method: (**a**) audio short-time zero-crossing rate; and (**b**) audio waveform. The vertical solid line is the start point of a call, the vertical dotted line is the end point of a call and the horizontal line is the threshold of the short-time zero-crossing rate.

In the short-time zero crossing rate method, the threshold of the short-time zero crossing rate ZCR was set to 10, the low threshold T1 of the duration T was set to 30 and the high threshold T2 was set to 110. The detection steps are shown in Figure 7, and the detection results are shown in Figure 8.

### 2.4. Audio Features

The chicks’ calls obtained through the above steps were in one-dimensional time-domain sequence. The one-dimensional sequence contains a large amount of information, but this information is hidden, so it is difficult to analyze the waveform directly [10]. Audio signals have many different audio features, such as spectrogram and MFCC. This paper selected three feature groups, spectrogram, cepstrogram and Logfbank+MFCC, for analysis. These features can express the time–frequency domain information of sound and achieved good results in various audio-processing problems.

#### 2.4.1. Spectrogram

Spectrogram is a feature that uses two-dimensional images to represent the time–frequency information of audio. Generally, its abscissa is time, its ordinate is frequency and its color indicates energy. Therefore, using a spectrogram can show the information of chicks’ calls in time and frequency domain [10,23]. The process of calculating the spectrogram was as follows:

(1) Perform Fourier transform separately for each frame of audio obtained by framing and windowing to obtain the spectrum of each frame and calculate the modulus of the spectrum to obtain the energy spectrum. Its calculation is shown in Equation (4) [24], where *N* is the number of sampling points contained in each frame of audio, *i* is the frame number, *y*(*n*) is the amplitude, and *E*(*n*) is the energy spectrum. Here, *N* was the window length, 1024.

(4) $$E_{i}\left(n\right)=\left|\overset{N-1}{\underset{n=0}{\sum }}y_{i}\left(n\right)e^{-j\omega n}\right|$$

(2) The energy spectrum was transformed from a linear scale to *dB* scale through logarithmic calculation, and the calculation is shown in Equation (5).

(5) $${EdB}_{i}\left(n\right)=10\times \log\left(E_{i}\left(n\right)\right)$$

**Figure 9:**
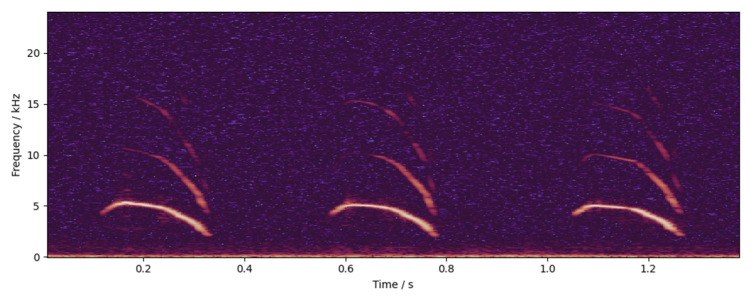
An example of spectrogram. The highlighted part in the figure is the high-energy point of the sound in the time–frequency domain. It can be seen that the pitch frequency of the chicken call is about 5000 Hz, while the frequency of the formant is higher, up to about 16,000 Hz.

(3) The *dB* scale energy spectrum was concatenated in time order to obtain the *dB* scale spectrogram of audio. An example of the spectrogram is shown in Figure 9.

#### 2.4.2. Cepstrogram

Cepstrogram is similar to spectrogram in that it uses two-dimensional images to represent the characteristics of audio time–frequency information. The difference between the cepstrogram and the spectrogram is that the composition of the spectrogram is short-time spectrum, while the composition of the cepstrogram is short-time cepstrum. The process to calculate the cepstrogram was as follows:

(1) Calculate the discrete cosine transform (DCT) of the logarithmic energy spectrum calculated by Equation (5). Its calculation is shown in Equation (6).

(6) $$Ceps_{i}\left(n\right)=\sqrt{\frac{2}{N}}\times \overset{N-1}{\underset{k=0}{\sum }}\left(EdB_{i}\left(k\right)\times \cos\left(\frac{\pi n\left(k-0.5\right)}{N}\right)\right)$$

**Figure 10:**
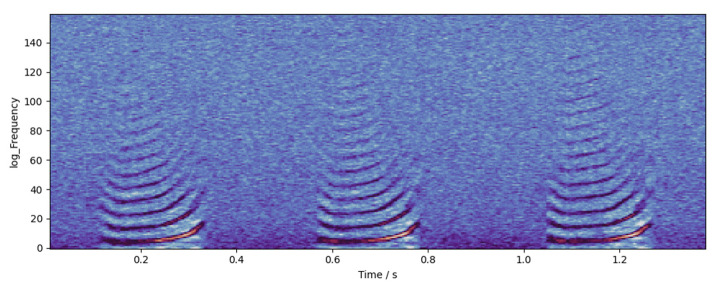
An example of cepstrogram. The highlighted part in the figure is the high-energy point of the sound in the time–frequency domain. After the logarithmic calculation, the fundamental frequency and formant of the sound are more prominent, which is conducive to observation and recognition.

(2) Take bits 5 to 165 of the cepstrum of each frame and concatenate them in time order to obtain the audio cepstrogram. The example of the cepstrogram is shown in Figure 10.

#### 2.4.3. Logfbank and Mel Frequency Cepstral Coefficients (MFCC)

MFCC is a widely used feature in automatic speech and speaker recognition. MFCC simulates the processing characteristics of human auditory perception, and its sensitivity to signals of different frequencies are different. By mapping the linear spectrum to the nonlinear Mel spectrum and reducing the dimension, the method has good recognition and anti-noise performances [25]. 

The relation between Mel frequency and linear frequency is shown in Equation (7) [26], where *f* is the linear frequency and *M*(*f*) is the corresponding Mel frequency.

(7) $$M\left(f\right)=2595\times \log\left(1+\frac{f}{700}\right)$$

**Figure 11:**
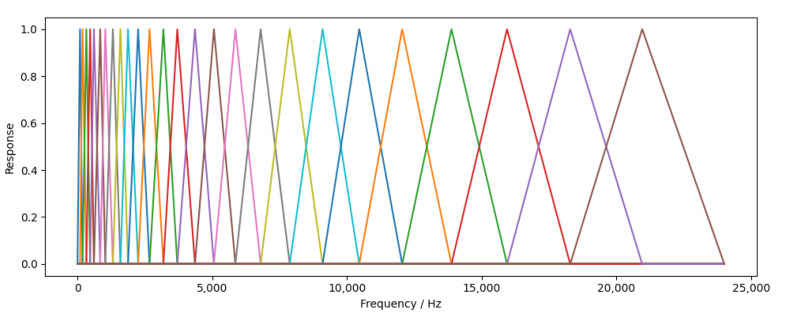
Mel filter bank.

During the calculation of MFCC, the dimension of data is reduced through Mel filter banks containing several filters, so as to greatly reduce the amount of data and retain as much important time–frequency characteristics of audio as possible. Twenty-six channel Mel filter banks were set in this experiment, and the schematic diagram is shown in Figure 11.

The process of calculating MFCC was as follows:

(1) According to Equation (4), calculate the linear scale energy spectrum *E_i_*(*n*) of each frame using the Fourier transform.
(2) Pass the energy spectrum through Mel filter bank to obtain the energy spectrum at the Mel frequency *MFE_i_*(*n*).
(3) Calculate the logarithm of Mel frequency energy spectrum and convert the linear scale into a logarithmic scale to obtain the log Mel frequency energy spectrum *log MFE_i_*(*n*), namely Logfbank.
(4) Calculate the discrete cosine transform (DCT) for the logarithmic energy spectrum *log MFE_i_*(*n*) to obtain the MFCC.

**Figure 12:**
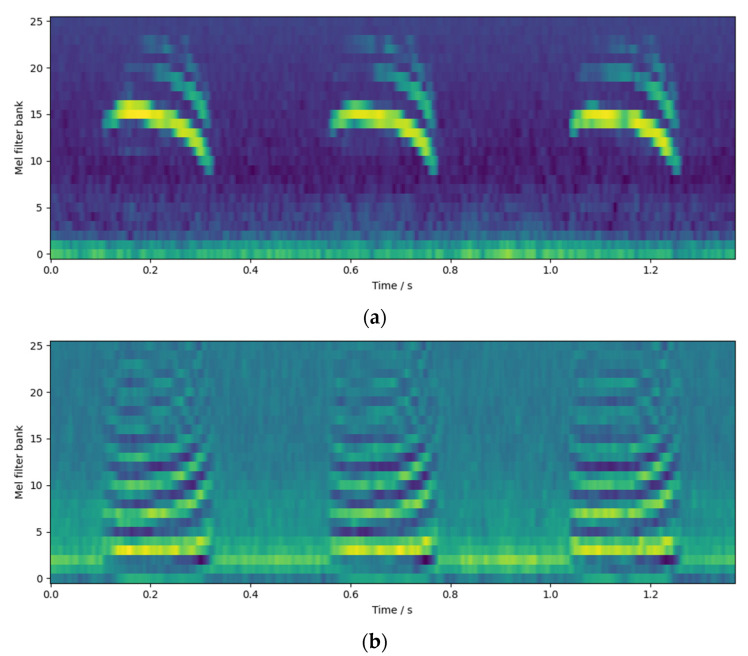
Example of feature group: (**a**) Logfbank; and (**b**) MFCC.

We concatenated Logfbank and MFCC to obtain a 52-dimensional combined audio feature. An example of this feature is shown in Figure 12. Figure 12a is Logfbank, which is equivalent to the dimensionality reduction in the spectrogram at the Mel frequency scale, and Figure 12b is MFCC, which is equivalent to the dimensionality reduction in the cepstrogram at the Mel frequency scale. The two features are spliced together to obtain a feature group.

### 2.5. Deep Learning Methods

After the signal processing and feature extraction, chick calls were transformed into two-dimensional audio features. However, these features cannot be recognized by human observation. Therefore, in order to classify chick calls, this paper used the data mining ability of the neural network to mine the deep information of these features and obtain the classification results. This paper selected and built a variety of neural networks, which were combined with the several features introduced above, and compared their results to find the optimal combination. The networks used in this paper include CNN, GRU, CRNN, TwoStream and ResNet-50.

CNN and RNN are the two most widely used neural networks. CNN can extract deep features from images, and RNN can extract features from time series. GRU is an improved RNN model. It only uses two gating units, reset gate and update gate. It has similar functions to LSTM, and the structure is simpler and lighter. Therefore, GRU has less parameters and computation than LSTM, and the training process is faster [27]. In order to recognize and detect the shape features and time series features of audio features at the same time, so as to improve the accuracy of the network, combining CNN with RNN, we obtained CRNN and TwoStream networks. ResNet-50 network was also used in this paper. ResNet-50 network is a variant of deep convolution neural network, which can greatly increase the network depth of CNN and restrain the degradation of the network through residual, inhibiting the gradient explosion and gradient disappearance of the network through batch normalization [28]. 

CNN has three convolution layers, and each convolution layer has 64 convolution cores. Maxpooling layers with the size of 3 × 3 are set between convolution layers. The tensor is then flattened through the flatten layer to obtain a one-dimensional sequence. GRU neural network has two GRU layers with 64 neurons in each layer. 

CRNN and TwoStream are the combination of CNN and GRU. The difference lies in the position of convolution part and recurrent part. CRNN means that the convolution part comes first and the recurrent part comes last. The convolution part is similar to the CNN network mentioned above. It has three convolution layers, and each convolution layer is equipped with 64 convolution cores with a size of 3 × 3. Maxpooling layers with the size of 3 × 3 are set between convolution layers. The convolution part is followed by the time flatten layer, which arranges the tensors in time order into a matrix composed of multiple one-dimensional arrays. The recurrent part is similar to the GRU network mentioned above, with two GRU layers, each with 64 neurons. 

TwoStream network divides the convolution part and the recurrent part into two branches that do not interfere with each other. The convolution branch is similar to the CNN network mentioned above, and the recurrent branch is similar to the GRU network mentioned above. The one-dimensional sequence obtained after the convolution branch passes through the flatten layer is concatenated with the output of the recurrent branch. 

**Figure 13:**
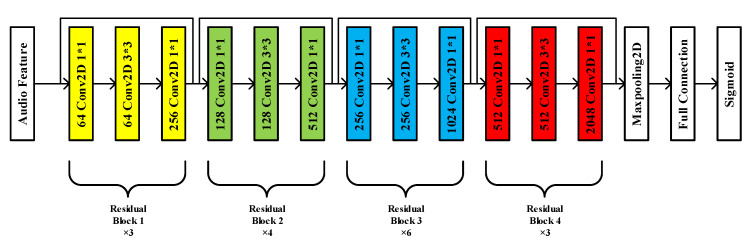
The structure of ResNet-50 neural network.

ResNet-50 network is pre-trained with “Imagenet” dataset. ResNet-50 network requires a matrix with three channels as input data, so the audio features are repeated three times in the channel dimension to obtain a matrix with three identical channels as input. ResNet-50 differs from the ordinary deep convolution neural network in that short-circuit mechanism is added between every three convolution layers to form residual learning. The main structure of ResNet-50 neural network includes four kinds of residual blocks. Residual block 1 mainly includes a convolution layer with 64 1 × 1 convolution cores, a convolution layer with 64 3 × 3 convolution cores and a convolution layer with 256 1 × 1 convolution cores. Residual block 2 mainly includes a convolution layer with 128 1 × 1 convolution cores, a convolution layer with 128 3 × 3 convolution cores and a convolution layer with 512 1 × 1 convolution cores. Residual block 3 mainly includes a convolution layer with 256 1 × 1 convolution cores, a convolution layer with 256 3 × 3 convolution cores and a convolution layer with 1024 1 × 1 convolution cores. Residual block 4 mainly includes a convolution layer with 512 1 × 1 convolution cores, a convolution layer with 12 3 × 3 convolution cores and a convolution layer with 2048 1 × 1 convolution cores. The head and tail of each residual block are short circuited to calculate the residual. The structure of ResNet-50 neural network is shown in Figure 13, which mainly includes 3 residual block 1, 4 residual block 2, 6 residual block 3 and 3 residual block 4.

The full connection layer is added to the ends of all neural networks in this paper, including a dense layer with 64 neurons and a dense layer with 32 neurons. The dropout between the two dense layers are 0.2. Finally, output the results through a sigmoid layer. All networks are trained using the Adam optimizer.

### 2.6. Experimental Design

In this study, the chicks’ sex corresponding to each call was detected. According to the comparison between the detected sex and the real sex, the accuracy was used as the main performance evaluation index. 

The ultimate goal of this study is to detect the sex of chicks through their calls. It can be seen from Figure 3 that the calls made by the same chick are highly similar, while the calls made by different chicks are very different. The shape of the spectrogram and the pitch frequency sequence of the calls made by the same chick are similar, and there is a big difference between the calls of different chicks in this respect. Therefore, it can be reasonably speculated that dispersing the calls from same chicks into the testing set and the training set will lead to false high testing results. 

Therefore, we randomly selected a part of all chick calls as the testing set. A more rigorous way is to randomly select a part of chicks in each variety and each sex in the same proportion as testing chicks, and use the majority voting strategy to detect the chicks’ sex through their calls, so as to obtain the final result. 

**Figure 14:**
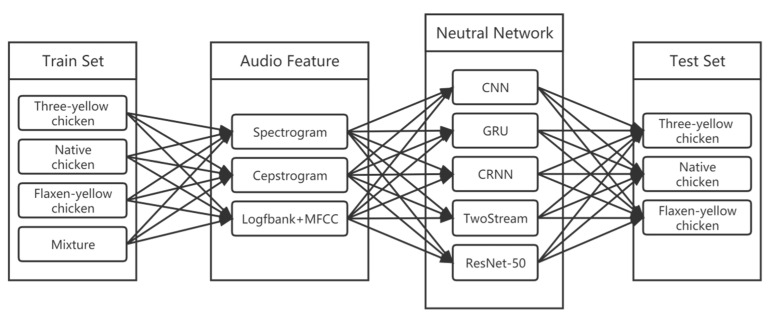
Structure of the cross-comparison experiment.

This study included three chick varieties, three audio features and five neural networks, and a cross-comparison experiment was designed. The experimental structure is shown in Figure 14, and the key points are shown below:

(1) In this experiment, while using the calls of each three variety of chicks alone for training, we also used a mixture of the calls of all three varieties of chicks for training.
(2) Four group of chick calls were combined with the three audio features to produce 12 training sets.
(3) A total of 12 training sets were input into 5 neural networks for training, and trained 60 networks.
(4) For each network, the calls of each of the three chicken varieties were used for testing, and 180 testing results were obtained.
(5) For each network, the test chicks of the three chicken varieties were used to detect the chicken sex based on the majority voting method and, finally, 180 detection results were obtained.

By comparing the results, we obtained the difference in the effect of sex detection using calls from different chicken varieties, and also selected the combination of audio features and neural networks with the best accuracy and generalization.

### 2.7. Dataset Construction

A total of 150 cocks and hens of three yellow chicken, 30 cocks and hens of native chicken and 100 cocks and hens of flaxen-yellow chicken were used in this study. Table 1 shows the number of chicks in each dataset and Table 2 shows the number of calls in each dataset.

For the three yellow chicken, 30 cocks and 30 hens were randomly selected, a total of 60 chicks were used as testing chicks, and the rest were used as training chicks. A total of 5000 cocks’ and 5000 hens’ calls were selected from all the calls of the training chicks, and a total of 10,000 calls were obtained for training. Then, 1000 cocks’ and 1000 hens’ calls were selected from the remaining calls, and a total of 2000 calls were used for validating. Finally, 2500 cocks’ and 2500 hens’ calls were selected from the calls of the testing chickens, and a total of 5000 calls were used for testing. Combined with three audio features, three training sets and three testing sets were formed. 

For the native chicken, 5 cocks and 5 hens were randomly selected, and a total of 10 chicks were used as testing chicks and the rest as training chicks. A total of 2500 cocks’ and 2500 hens’ calls were selected from all the calls of the training chicks, and a total of 5000 calls were obtained for training. Then, 500 cocks’ and 500 hens’ calls were selected from the remaining calls, with a total of 1000 calls being used for validation. Finally, 1500 cocks’ and 1500 hens’ calls were selected from the calls of the testing chickens, and a total of 3000 calls were used for testing. Combined with three audio features, three training sets and three testing sets were formed. 

For the flaxen-yellow chicken, 15 cocks and 15 hens were randomly selected, and a total of 30 chicks were used as testing chicks and the rest as training chicks. A total of 5000 cocks’ and 5000 hens’ calls were selected from all the calls of the training chicks and, a total of 10,000 calls were obtained for training. Then, 1000 cocks’ and 1000 hens’ calls were selected from the remaining calls, and a total of 2000 calls were used for validation. Finally, 2500 cocks’ and 2500 hens’ calls were selected from the calls of the testing chickens, and a total of 5000 calls were used for testing. Combined with three audio features, three training sets and three testing sets were formed. 

Finally, 2500 cocks’ and hens’ calls were randomly selected from the three yellow chicks, 2500 cocks’ and hens’ calls were randomly selected from the native training chicks and 2500 cocks’ and hens’ calls were randomly selected from the flaxen-yellow training chicks, with a total of 15,000 calls being used for training. Then, 500 cocks’ and hens’ calls were randomly selected from the remaining calls of the three yellow training chicks, 500 cocks’ and hens’ calls were randomly selected from the remaining calls of the native training chicks and 500 cocks’ and hens’ calls were randomly selected from the remaining calls of the flaxen-yellow training chicks, with a total of 3000 calls being used for validation. Combined with three audio features, three training sets were formed.

## 3. Results and Discussions

### 3.1. Sex detection of Vocalization

**Figure 15:**
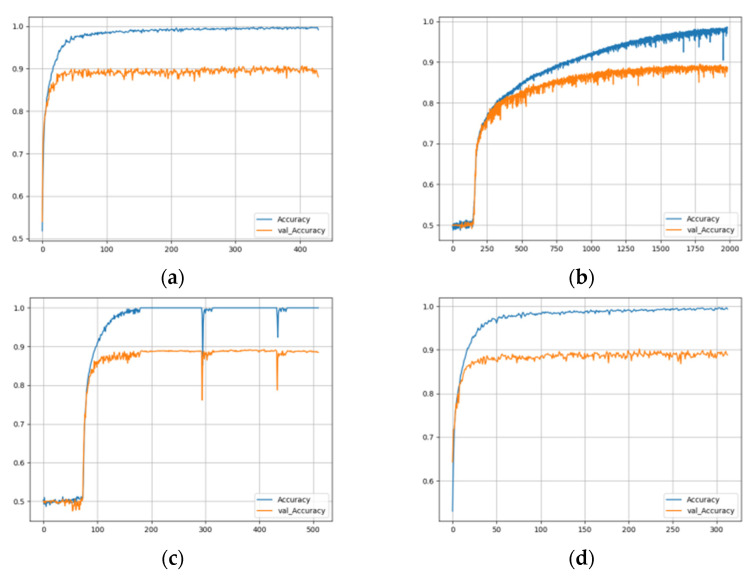

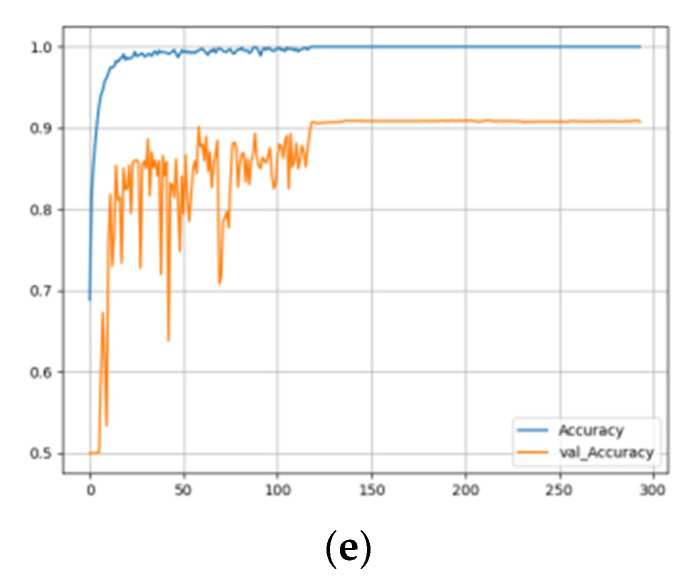
Training history: (**a**) CNN; (**b**) GRU; (**c**) CRNN; (**d**) TwoStream; and (**e**) ResNet-50.

In the training process, the EarlyStoping strategy was adopted. We monitored the validating accuracy and, if the validating accuracy did not increase in 100 training epochs, the training was stopped, and the epoch with the highest validating accuracy in these 100 epochs was taken as the final result of the training. For example, the record of training five neural networks using the three yellow chicken’s call and MFCC+Logfbank audio features is shown in Figure 15. From the training records of each model, it can be observed that when each network tends to be stable and the validating accuracy can reach about 90%, but the training time of each network is different. CNN and TwoStream networks are the fastest, gradually becoming stable at about 100 epochs. ResNet-50 network needs 150 epochs, CRNN network needs 200 epochs, and GRU network has the slowest training speed, needing nearly 2000 epochs to stabilize gradually.

**Figure 16:**
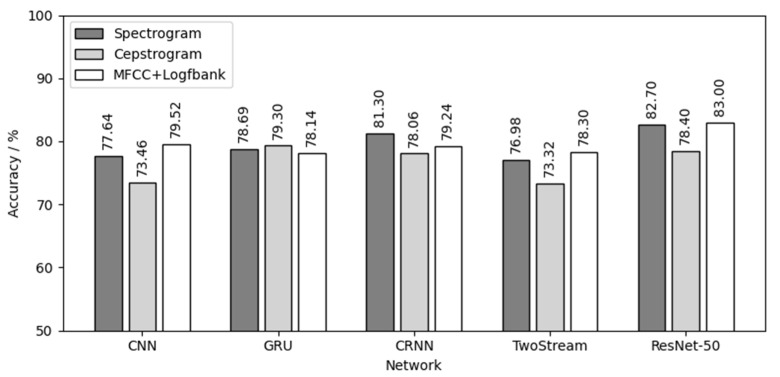
Three yellow chick calls for both training and testing.

Then, 60 models were tested by three testing sets of three chick varieties, and 180 test results were obtained. Figure 16 shows the result of using three yellow chicks’ calls for both training and testing. For the three yellow chicks, ResNet-50 had the best effect in the five networks with an average testing accuracy of 81.37%. MFCC+Logfbank has the best effect in the three audio features with an average testing accuracy of 79.64%. The combination of MFCC+Logfbank audio feature and ResNet-50 network achieved the best results, with the highest testing accuracy of 83%.

**Figure 17:**
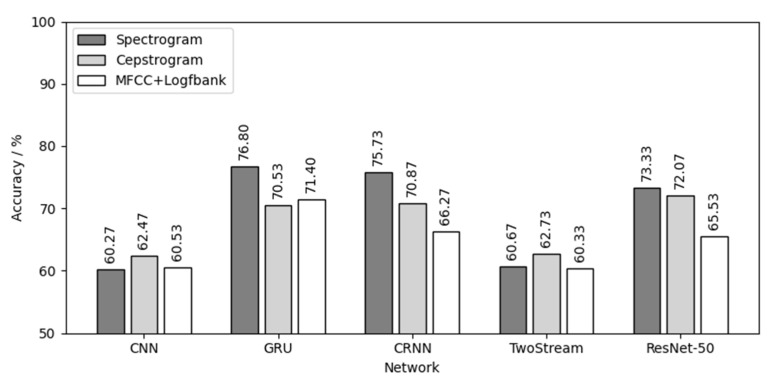
Native chick calls for both training and testing.

Figure 17 is the result of using the native chick calls for both training and testing. For the native chicks, GRU had the best effect in the five networks, with an average testing accuracy of 72.91%. The spectrogram had the best effect in the three audio features, with an average testing accuracy of 69.36%. The combination of spectrogram audio feature and GRU network achieved the best results, with the highest testing accuracy of 76.8%.

**Figure 18:**
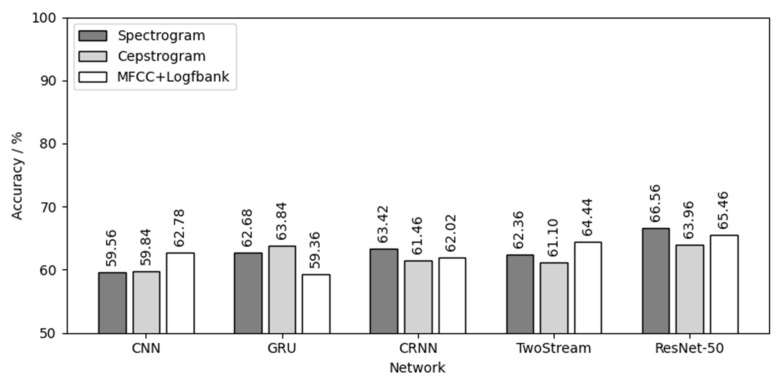
Flaxen-yellow chick calls for both training and testing.

Figure 18 is the result of using flaxen-yellow chicks’ calls for both training and testing. For the flaxen-yellow chicks, ResNet-50 had the best effect in the five networks, with an average testing accuracy of 65.33%. The spectrogram had the best effect in the three audio features, with an average testing accuracy of 62.92%. The combination of spectrogram audio feature and ResNet-50 network achieved the best results, with the highest testing accuracy of 66.56%.

Comparing the results of Figure 16, Figure 17 and Figure 18, the following conclusions can be drawn:

(1) Different chick varieties have different sex separability in their calls. The three yellow chicks had the highest test accuracy, which indicates that the difference of vocalizations between different sexes of three yellow chicks was the most obvious and clear, and the separability was good. Second was the native chicks and the flaxen-yellow chicks were the worst, indicating that the differences in vocalizations between the two sexes were not obvious, not clear or even that there might be crossover, that is, poor separability, resulting in a low test accuracy.
(2) Among the five neutral networks, the ResNet-50 network had the best overall performance, followed by the CRNN network, because the ResNet-50 network has the largest depth, can extract more deep features and is also pre-trained by the “Imagenet” dataset. CRNN network can analyze the time series characteristics of chick calls as time-varying signals on the basis of CNN, which can also increase the depth of the network, and thus achieve good results. CNN, GRU and TwoStream have fewer layers and less depth than CRNN and ResNet-50, causing poor results.
(3) Among the three audio features, the spectrogram and MFCC+Logfbank had the best overall performance, having different classification capabilities for different chick varieties. The calculation, transformation and compression of the original audio during the calculation of spectrogram were the lowest among the three features, and the data size of spectrogram was the largest. Therefore, it retained the most audio features, which is helpful to achieve better results in the classification of chick calls of poor separability, such as native chicks and flaxen-yellow chicks. MFCC+Logfbank had the least amount of data, which reduces the size of data greatly while retaining as many important features as possible. When classifying the dataset with good separability, such as the chick calls of the three yellow chickens, it can reduce the over-fitting of the network and improve the robustness of the network, so it can obtain better results in the training and testing of the three yellow chick calls.

Table 3 shows the testing results of using calls of chicks of different varieties for training and testing, where TC is three yellow chicken, NC is native chicken and FC is flaxen-yellow chicken. As shown in Table 3, the network trained with calls from a single chicken variety was very ineffective when tested with calls from other varieties of chicks. Most of the results are around 50%. By comparing these results with those of Figure 16, Figure 17 and Figure 18, it can be inferred that there was a large diversity between the sex differences of chick calls of different varieties, meaning that the network trained with a single variety of chick calls cannot be used for calls from other varieties of chicks.

Table 4 presents the results obtained by using a mixture of calls from all three varieties of chicks for training and using calls from each three variety of chicks for testing. The following conclusions can be drawn from the results in Table 4:

(1) When a mixture of calls from three varieties of chicks were used as training sets, the results of the tests were significantly lower than those of Figure 16, Figure 17 and Figure 18 using calls from a single identical variety of chicks for training and testing. The reason may be that there was a large diversity between the sex differences of chick calls of different varieties. Mixing different varieties of chick calls for training hinders the neural network from finding truly effective sex characteristics. Therefore, it can be concluded that, in order to classify the calls of different sexes more effectively, the best method is to train the network with the calls of a single, identical chicken variety.
(2) When using the same number of calls from three different varieties of chicks as the training set, the three yellow chick calls, used as a test, still achieves better results, which also verifies the inference that the three yellow chicken calls have better sex separability.

**Table 4:** Results of using a mixture of calls from the three varieties of chicks for training and using calls from the three varieties of chicks for testing.

Audio Feature	Testing Variety	CNN	GRU	CRNN	TwoStream	ResNet-50
Spectrogram	TC	75.64%	72.92%	75.38%	74.70%	49.88%
	NC	61.07%	70.80%	69.87%	57.33%	47.93%
	FC	60.20%	58.36%	59.22%	58.96%	45.48%
Cepstrogram	TC	71.82%	68.16%	67.18%	68.78%	51.38%
	NC	57.20%	58.07%	58.80%	58.80%	42.60%
	FC	58.04%	57.38%	58.88%	57.90%	48.64%
MFCC+Logfbank	TC	74.62%	72.98%	72.82%	73.10%	60.90%
	NC	60.60%	70.00%	62.93%	57.53%	48.87%
	FC	61.12%	57.96%	57.90%	62.26%	49.48%

### 3.2. Sex Detection of Chicks

After the training and testing of all networks, in order to verify the performance of the network in detecting chick sex by chick calls, this paper used the majority voting method to detect chick sex. In the 2 min audio sequences of each chick, there were some silence and noise pieces, so 30 s of effective audio was cut from each chick’s 2 min audio for sex detection. The number of calls of different chicks within 30 s ranged from 40 to 80. According to previous research [19], 41 calls proved to be applicable to the majority voting method for chick sex detection. Therefore, 41 calls per chick were used in this research. For each chick, 41 calls were used for sex detection, and the results appearing more times in 41 results were used as the final detection result of the sex of the chick. This method can exclude erroneous judgements and significantly improves the detection accuracy. A total of 180 models were obtained in Section 3.1, so the detection of chick sexes would also yield 180 results.

**Figure 19:**
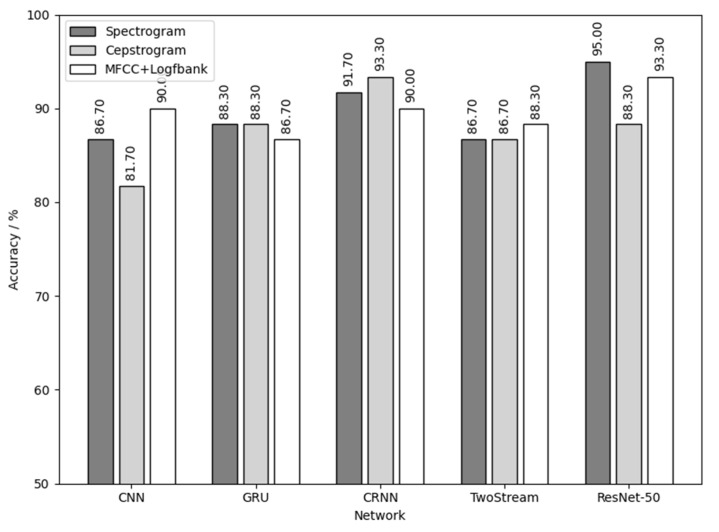
Three yellow chick calls used for training and to detect the sex of the three yellow chicks.

Figure 19 shows the result of using the three yellow chick calls for training and detecting the sex of the three yellow chicks. As shown in Figure 19, the combination of spectrogram audio feature and ResNet-50 network achieves the best results with the sex detection accuracy of 95%. CRNN network also achieves good results, with a sex detection accuracy of over 90%.

**Figure 20:**
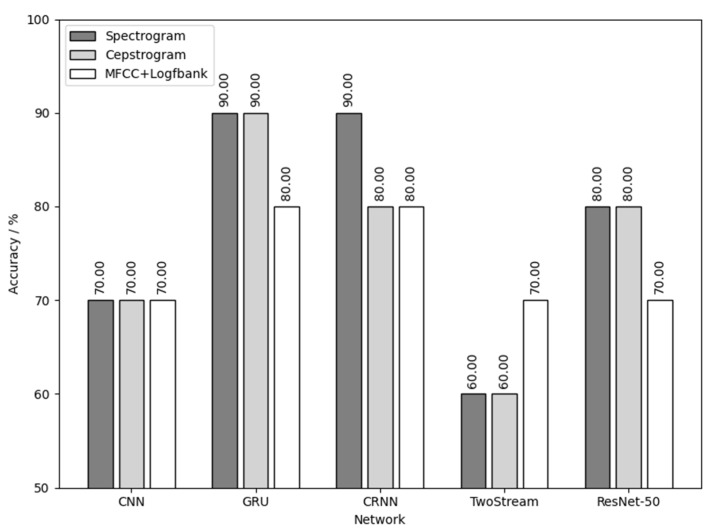
Native chick calls used for training and to detect the sex of native chicks.

Figure 20 is the result of using native chick calls for training and detecting the sex of the native chicks. As shown in Figure 20, the highest sex detection accuracy was 90%. The data fluctuated too much due to the small number of native chicks, but the results are still persuasive combined with those of Figure 17. Additional experiments can then be performed to expand the dataset of the native chicks to improve the detection results.

**Figure 21:**
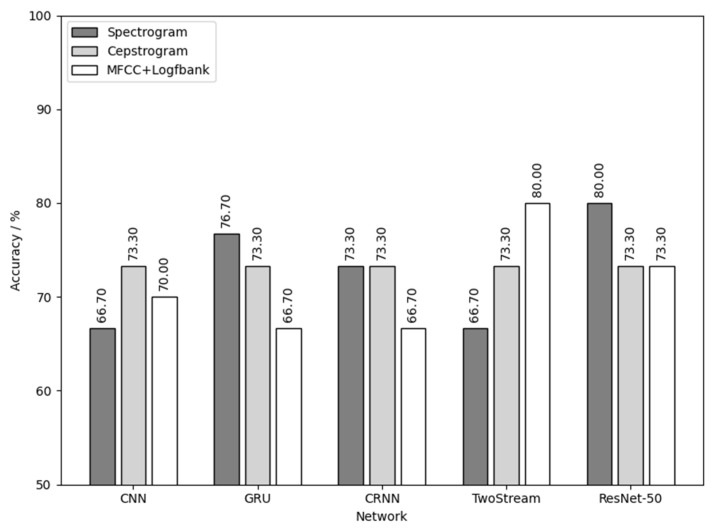
Flaxen-yellow chick calls used for training and to detect the sex of the flaxen-yellow chicks.

Figure 21 is the result of using flaxen-yellow chick calls for training and detecting the sex of flaxen-yellow chicks. As shown in Figure 21, the combination of MFCC+Logfbank audio feature and TwoStream network and the combination of spectrogram audio feature and ResNet-50 network had the best results, with a sex detection accuracy of 80%.

Table 5 shows the result of using a mixture of calls from all three varieties of chicks for training and performing sex detection on each of the three varieties of chicks. From Table 5, we can see that the GRU network obtains the best results in chick sex detection when using the mixed training set. At the same time, among the three varieties of chicks, the three yellow chicks had the highest accuracy of sex detection, reaching 90%.

Comprehensively analyzing the results in Figure 19, Figure 20 and Figure 21 and Table 5, it can be concluded that the results of chick sex detection are highly correlated with the testing results in Section 3.1, and were generally improved. Among them, the three yellow chick had the best sex detection results, the native chick had the second best and the flaxen-yellow chick had the worst. This verifies the inference made in Section 3.1, which reflects the sex separability differences of calls of three varieties of chickens.

Table 6 shows the time required for the combination of three audio features and five neural networks to complete the detection of 41 calls of a three-yellow chicken. The CPU of the equipment used in the test was Intel Core i5-12400F and the GPU was RTX3060-12G. From Table 6, it can be seen that, among the three audio features, MFCC+Logfbank is the audio feature that takes the least time, cepstrogram is the second and spectrum is the most time-consuming. The reason is that the size of the three features is different. The size of spectrogram of a call is 110 × 513, the size of cepstrogram is 110 × 160 and the size of MFCC+Logfbank is 110 × 52. The size of the features directly affects the amount of network parameters and the speed of calculation. Among the five kinds of neural networks, ResNet-50 takes longer than the other networks because of its great depth and parameter quantity. GRU and CNN take less time than the other neural networks, but with the increase in features’ size, CNN takes more time than GRU, because with the increase in features’ size, the parameters of CNN also increase. Combined with the analysis of Figure 15, in general, GRU needs the most training epochs, but the time required for chick sex detection is the lowest. 

### 3.3. Discussion

Pereira demonstrated, by Student’s *t*-test and Fisher’s test, that the frequency of the second formant and the distance between the formants are highly correlated with the chick sex and can thus be used to detect chick sex [16]. However, this method has not actually been used to detect chick sex in the study, so it is unclear whether the method is effective. Chen et al. obtained an average accuracy of 85.41% by using pitch frequency sequences, Hidden Markov models and major voting methods to detect chick sex by calls [17]. However, only 30 chickens were used in the experiment, thus making the results not convincing. Moreover, the paper did not specify the breed of chick and recording conditions. Additionally, the accuracy of 85.41% was still far from effective. Sadeghi and Banakar used a Support Vector Machine (SVM) with Gauss Radial Basis Function (GRBF) to classify the audio characteristics of chick calls in time, frequency and time–frequency domains with accuracies of 68.51%, 70.37% and 90.74%, respectively [18]. The accuracy of 90.74% is considerable, but there are still some drawbacks. First, the number of 360 calls from cocks and hens was still insufficient. Second, 30% of 360 calls, or 108 calls, were randomly taken out for testing. As a result, the calls of some chicks were divided into training and testing sets at the same time. The key to testing a classifier is to use data that the classifier has not seen during training. However, due to the high similarity of the calls from the same chick, some of the calls in the test set were actually seen by the classifier. This is closer to the concept of validation set in deep learning, resulting in false high test results. This is also a problem in the experiment conducted by Chen et al. [17]. Cuan et al. selected a deep learning method, using CNN, LSTM and GRU as classifiers; the highest test accuracy was 76.15% and the highest accuracy using the majority voting method was 91.25% [19]. The study used a total of 300 chickens and 15,000 calls, and the data were sufficient. The study also strictly distinguished between the training and test chicks so that calls from the same chick did not appear in both the training and test sets. All the sounds the model sees during the test are unfamiliar, which greatly improves the strictness of the test. The only problem with this study is that no extensive studies have been carried out on other breeds of chicks to verify the universality of this method.

On the basis of the experiments carried out by Cuan et al., two breeds of chicks, native chicks and flaxen-yellow chicks, were added to the experiment in order to verify the universality of the method. After finding that the sex detection results of the native chicks and flaxen-yellow chicks were not good, considering that the reason may be the separability difference of the chick call dataset itself, other kinds of audio features and neural networks were added to the comparison and experiment. Comparing the results of different combinations of audio features and neural networks on different breeds of chicks, we can see that the sex detection method of chick based on calls cannot be applied to all breeds of chicks. The reason could be that the sex differences in the calls of different breeds of chickens are different, which is reflected in the differences in separability of the dataset. Three yellow chicks differ significantly in the sex characteristics of their calls and have fewer crossovers between different sexes, which makes it easy to distinguish, whereas native chicks and flaxen-yellow chicks differ less and have more crossovers between different sexes, making it difficult to distinguish.

The reason for the different results in the different methods of detecting the sex of same breed of chick is also related to the dataset itself. Different kinds of audio features contain different information and their weights. Their sizes are different as well. The training direction, focus and data-mining ability of different neural networks are also different in the training process, which leads to different results. Overall, the combination of the deepest neural network ResNet-50 and the largest audio feature spectrogram has a better ability to detect sex, which offered the best result in the sex detection of the three yellow chicks and flaxen-yellow chicks, and obtained the second best result in the sex detection of the native chicks.

The different sex detection accuracy of different breeds proves that the sex detection method based on calls cannot be applied to all breeds of chicks. Different breeds of chicks have different sex characteristics in their calls. Every new breed of chick needs to be tested separately to determine whether the differences in the sex characteristics of the calls are significant, and whether the method based on the calls is applicable, before the method is applied to it.

There is still much work that needs to be conducted in the future. More breeds of chicks need to be tested to determine the range of application of this method. More call data need to be collected to expand the dataset and achieve a higher accuracy. Different combinations of audio features and neural networks need to be compared, and the best combination should be selected considering accuracy, calculations required and time consumed. An automated process and its supporting equipment need to be designed in order to make this sex detection method fully practical and automatic.

## 4. Conclusions

In this paper, a method to detect the sex of chicks according to their calls was designed. The cross-comparison experiments were carried out on three varieties of chicks, three kinds of audio feature and five kinds of neural networks. The results show that the ResNet-50 neural network trained with MFCC+Logfbank audio feature of the three yellow chick calls had the highest test accuracy of 83% when testing the three yellow chick calls. When the majority voting method was used to detect the sex of chicks, the ResNet-50 neural network trained with the spectrogram audio features of the three yellow chick calls had the highest accuracy of 95% when detecting the three yellow chick sex. At the same time, the results of the cross-comparison experiments also show that there is a large diversity between the sex differences of chicks of different varieties. When detecting the sex of chicks of a similar variety to that of the training chicks, the method can obtain better results, while detecting the sex of chicks of other varieties significantly reduces the detection accuracy. Only using calls from the same variety of chick for training and detecting can achieve the best results.

In general, the method proposed in this paper can help to automatically detect the sex of chicks according to their calls, which can reduce the manual workload and requirement of professional skills in sex detection. The cross-comparison experiment of multiple chicken varieties, multiple audio features and multiple neural networks conducted in this paper also provides further perspectives for the chick sex detection method based on the call and provides help and guidance for other researchers’ future research.

## Figures and Tables

**Table 1:** The number of chicks in each dataset.

Variety	Dataset	Sex	Number of Chicks
Three yellow chicken	Training	Cock	120
		Hen	120
	Test	Cock	30
		Hen	30
Native chicken	Training	Cock	25
		Hen	25
	Test	Cock	5
		Hen	5
Flaxen-yellow chicken	Training	Cock	85
		Hen	85
	Test	Cock	15
		Hen	15

**Table 2:** The number of calls in each dataset.

Variety	Dataset	Sex	Number of Calls
Three yellow chicken	Training	Cock	5000
		Hen	5000
	Validation	Cock	1000
		Hen	1000
	Test	Cock	2500
		Hen	2500
Native chicken	Training	Cock	2500
		Hen	2500
	Validation	Cock	500
		Hen	500
	Test	Cock	1500
		Hen	1500
Flaxen-yellow chicken	Training	Cock	5000
		Hen	5000
	Validation	Cock	1000
		Hen	1000
	Test	Cock	2500
		Hen	2500

**Table 3:** Testing results of using calls of chicks of different varieties for training and testing.

Training Variety	Audio Feature	Testing Variety	CNN	GRU	CRNN	TwoStream	ResNet50
TC	Spectrogram	NC	52.80%	46.20%	48.87%	56.87%	48.27%
		FC	53.54%	56.60%	53.04%	54.82%	55.16%
	Cepstrogram	NC	47.87%	50.40%	49.13%	54.20%	49.53%
		FC	57.38%	54.86%	58.62%	57.34%	56.26%
	MFCC+ Logfbank	NC	48.47%	56.40%	55.93%	49.33%	49.60%
		FC	56.34%	58.12%	55.96%	56.20%	55.14%
NC	Spectrogram	TC	55.36%	49.68%	58.22%	54.42%	53.04%
		FC	55.84%	45.38%	51.34%	54.12%	49.16%
	Cepstrogram	TC	55.26%	53.30%	56.32%	55.54%	51.82%
		FC	51.00%	50.50%	48.70%	51.46%	50.08%
	MFCC+ Logfbank	TC	55.08%	49.78%	50.20%	53.92%	53.84%
		FC	48.24%	48.06%	47.70%	50.90%	48.54%
FC	Spectrogram	TC	65.40%	65.62%	66.68%	65.48%	57.46%
		NC	48.73%	49.47%	43.20%	48.33%	53.33%
	Cepstrogram	TC	62.62%	63.24%	64.30%	61.82%	66.96%
		NC	45.27%	51.07%	43.13%	43.53%	50.40%
	MFCC+ Logfbank	TC	61.98%	64.88%	62.28%	61.98%	64.10%
		NC	50.47%	54.13%	42.60%	48.00%	48.33%

**Table 5:** Result of using a mixture of calls from the three varieties of chickens for training and performing sex detection on each of the 3 varieties of chickens.

Audio Feature	Detecting Variety	CNN	GRU	CRNN	TwoStream	ResNet-50
Spectrogram	TC	86.70%	88.30%	90.00%	85.00%	50.00%
	NC	60.00%	80.00%	80.00%	60.00%	50.00%
	FC	66.70%	73.30%	70.00%	66.70%	46.70%
Cepstrogram	TC	85.00%	78.30%	73.00%	78.30%	50.00%
	NC	50.00%	70.00%	60.00%	60.00%	40.00%
	FC	60.00%	66.70%	60.00%	66.70%	53.30%
MFCC+Logfbank	TC	85.00%	78.30%	83.30%	88.30%	65.00%
	NC	60.00%	90.00%	70.00%	60.00%	40.00%
	FC	73.30%	60.00%	70.00%	76.70%	50.00%

**Table 6:** The time required for the combination of three audio features and five neural networks to complete the detection of 41 calls of a three-yellow chicken.

	CNN	GRU	CRNN	TwoStream	ResNet-50
Spectrogram	0.094 s	0.075 s	0.108 s	0.115 s	0.213 s
Cepstrogram	0.069 s	0.069 s	0.081 s	0.088 s	0.144 s
MFCC + Logfbank	0.062 s	0.067 s	0.074 s	0.081 s	0.129 s

## Data Availability

Data sharing is not applicable to this article.
